# Plasma extracellular vesicle sampling from glioblastoma demonstrates a small RNA signature indicative of disease and identifies lncRNA *RPPH1* as a biomarker

**DOI:** 10.1093/noajnl/vdaf273

**Published:** 2026-01-07

**Authors:** Jae Ho Han, Gabriel Wajnberg, Kathleen M Attwood, Lindsay Noiles, Brandon Hannay, Robert Cormier, Simi Chacko, Maya Willms, Andrea L O Hebb, Mary V Macneil, Matthias H Schmidt, Sidney E Croul, Adrienne C Weeks, Jeremy W Roy

**Affiliations:** Division of Neurosurgery, Dalhousie University, Halifax; Beatrice Hunter Cancer Research Institute, Halifax; Atlantic Cancer Research Institute, Moncton; Division of Neurosurgery, Dalhousie University, Halifax; Atlantic Cancer Research Institute, Moncton; Atlantic Cancer Research Institute, Moncton; Atlantic Cancer Research Institute, Moncton; Atlantic Cancer Research Institute, Moncton; Department of Medical Neuroscience, Dalhousie University, Halifax; Division of Neurosurgery, Dalhousie University, Halifax; Division of Medical Oncology, Dalhousie University, Halifax; Department of Diagnostic Radiology, Dalhousie University, Halifax; Department of Pathology, Dalhousie University, Halifax; Beatrice Hunter Cancer Research Institute, Halifax; Division of Neurosurgery, Dalhousie University, Halifax; Beatrice Hunter Cancer Research Institute, Halifax; Atlantic Cancer Research Institute, Moncton; Beatrice Hunter Cancer Research Institute, Halifax

**Keywords:** biomarkers, extracellular vesicles, glioblastoma, LncRNA *RPPH1*, small RNA sequencing

## Abstract

**Background:**

Glioblastoma (GBM) and cells of the tumour microenvironment (TME) secrete extracellular vesicles (EVs) into the plasma that contain genetic and protein cargo, which function in paracrine signaling. Isolation of these EVs and their cargo from plasma could lead to a simplistic tool that can inform on diagnosis and disease course of GBM.

**Methods:**

In the present study, plasma EVs were captured utilizing a peptide affinity method (Vn96 peptide) from GBM patients and normal controls followed by next generation sequencing to define a small RNA (sRNA) signature unique to GBM.

**Results:**

Over 750 differentially expressed sRNA (miRNA, snoRNA, lncRNA, tRNA, mRNA fragments and non-annotated regions) were identified between GBM and controls. MiEAA 2.0 pathway analysis of the miRNA in the sRNA signature revealed miRNA highly enriched in both EV and GBM pathways demonstrating the validity of results in capturing a signal from the TME. Also revealed were several novel GBM plasma EV sRNA biomarkers including lncRNA RPPH1 (Ribonuclease P Component H1), RNY4 (Ro60-Associated Y4) and RNY5 (Ro60-Associated Y5). Furthermore, in paired longitudinal patient plasma sampling, RPPH1 informed on surgical resection (decreased on resection) and importantly, RPPH1 increased again on clinically defined progression.

**Conclusions:**

The present provides preliminary data that support the continued investigation of plasma EV sRNA sampling (and particularly RPPH1) as part of a multi-pronged approach to GBM diagnosis and disease course surveillance.

Key Points

- Diagnosis of GBM is enabled by sampling plasma sRNA EV cargo.
- lncRNA *RPPH1* contained in EVs may act as a barometer of GBM disease.

Importance of the Study

Liquid biopsy of plasma EVs is a safe, less invasive approach that can be utilized to monitor GBM disease progression in the future. We have identified *RPPH1* as a sensitive marker of GBM disease from diagnosis, post-surgery, and then progression. *RPPH1* should be studied in a prospective manner to determine if it can aid in the diagnosis of GBM recurrence. More interestingly, given that *RPPH1* has specific biological function, increased *RPPH1* in GBM EVs and other cancers necessitates further testing to determine if RPPH1 demonstrates therapeutic potential in addition to its role as a biomarker.

Glioblastoma (GBM) is the most common primary malignant brain tumour, definitively diagnosed with invasive surgery.^1^^,^^2^ The 2021 World Health Organization (WHO) defines GBM as isocitrate dehydrogenase wild-type (*IDH*-*WT*), this is in contrast to Grade 4 astrocytoma tumours which are a distinct glioma subtype that demonstrate IDH mutations.^3^^,^^4^ The *IDH* mutation defines 2 subsets of tumours that differ in terms of patient demographic, genetic and prognostic factors. This study was restricted to GBM with intact IDH. GBMs are universally fatal (median survival 14-20 months) despite multimodal therapy (surgical resection, radiation, chemotherapy and tumour-treating fields).^5-7^ GBM progression is the rule, as these tumours are proficient at adapting to a hostile microenvironment and co-opting surrounding normal brain cells (astrocytes and neurons), immune cells (eg, microglia, monocytes and macrophages), and endothelial cells to promote survival and progression.^8-10^

Recent evidence suggests that several modalities of cell-to-cell communication between GBM cells and those of the tumour microenvironment (TME) enable pro-tumorigenic ­features.^8^^,^^11-14^ Secretion of membrane-bound extracellular vesicles (EVs) serve as an efficient means of bidirectional communication between GBM and cells of the TME to mediate autocrine/paracrine signaling.^10^^,^^13-24^ For example, GBM cells release EVs containing pro-angiogenic proteins such as vascular endothelial growth factor isoforms A and C, or fibroblast growth factor that target the surrounding endothelium and promote tumour vascularity.^25-30^ Alternatively, EVs released by normal astrocytes containing tumour-suppressive cargo have been shown to inhibit growth of GBM.^12^

EVs (ranging in size from 30 nm to 1000 nm) are secreted into all biofluids such as blood and cerebrospinal fluid (CSF) and are subdivided according to their size and subcellular origin (exosomes, microvesicles and apoptotic bodies).^22^^,^^31^^,^^32^ While EVs are produced by almost all cell-types,^33^ malignant GBM cancer cells have been shown to increase production of EVs and their secretion into plasma.^34^^,^^35^ Despite the blood-brain barrier, EVs derived from GBM tumours have been detected in the plasma, most notably in the well-designed experiment using surgically resected tissue and plasma collected from patients treated with 5-aminolevulinic acid.^36^ Thus, the isolation of plasma EVs and elucidation of their cargo is an appealing non-invasive methodology (liquid biopsy) for informing on GBM. Numerous methods of EV isolation have been described in the literature to analyze various cancer pathologies.^36-39^ The Vn96 peptide is a peptide affinity capture method developed for the capture of EVs in clinically relevant biofluids.^19-21^^,^^34^^,^^40-43^ By design, Vn96 peptide has a propensity to bind heat shock proteins (HSPs), which are abundant on EVs and capturing HSP-containing EVs, analyzing their cargo, can differentiate differing contextual clinically relevant information, such as invasive phenotype.^21^ Recently, vesicular Hsp70 levels were shown to be significantly increased in the plasma of GBM patients, therefore utilizing an EV capture technology that might take advantage of this would be beneficial.^44^ Numerous studies have shown captured Vn96-EVs from plasma contain canonical EV markers, and Vn96 has been utilized for the discovery of EV cargo-biomarkers in amyotrophic lateral sclerosis^41^ and cancers, such as prostate,^45^ lung,^46^ pediatric B-lymphoblastic leukemia^47^ and pancreatic ductal adenocarcinoma.^48^

Non-coding RNAs (such as miRNA, snoRNA, lncRNA, piRNA and others) contained in EVs of GBM plasma and those of cancers outside of the brain, have already been shown to be effective forms of tumour-associated cell-to-cell communication.^28^^,^^30^^,^^36^^,^^49-52^ For example, the long non-coding RNA (lncRNA) ribonuclease P RNA component H1 (*RPPH1*) has been shown to play an important role in multiple cancers (eg, lung, colorectal and breast cancer).^53-55^ Moreover, *RPPH1* contained in EVs isolated from colon cancer has been shown to regulate M2 polarization in macrophages of the TME, promoting proliferation and metastases of colon cancer cells.^53^ In GBM patient plasma-EVs, *MALAT1* was found to promote tumour proliferation and chemoresistance.^56^ GBM angiogenesis was mediated by EV-associated *CCAT2* or *HIF1A-AS*,^57^^,^^58^ and immune regulation of the GBM TME by EV *MROCKI* or *LNCARSR.*^59^^,^^60^ The abnormal expression levels of various miRNA isolated from the EVs of GBM patient plasma have been found to mediate tumour aggressiveness and correlate with overall survival. Low levels of miR-485-3p were shown to correlate with significantly worse survival in GBM patients.^61^ Angiogenesis was regulated by EV miR-1, miR-9 or miR-148a-3p,^62-64^ and TME immune regulation by miR-451, miR-21 and miR-138.^18^^,^^65^ EV miR-1238, miR-135b or miR-151a were found to be critical in acquired treatment resistance.^66-68^ These and other studies establish an ever-growing list implicating EVs and their small RNA (sRNA) cargo in many aspects of GBM tumorigenesis and cancer as a whole.

We aim to demonstrate that GBM-associated plasma-EVs obtained with peptide affinity capture can serve as promising non-invasive biomarkers of disease with the potential to interrogate pro-tumour crosstalk.^10^^,^^22^^,^^25^^,^^38^^,^^69-74^ For the first time in GBM we have utilized peptide affinity capture of plasma-EVs to determine a sRNA signature that can inform on GBM in patient samples pre- and post-surgery. Our sRNA signature recapitulates previous data demonstrating the validity of our method and adds novel potential sRNA biomarkers. These data can be used to aid in the establishment of biomarkers of disease and potential therapeutic targets in the future.

## Methods

### Patient and Sample Collection

Ethical approval was obtained from the Research Ethics Board of Nova Scotia Health (REB#1023343). Adult patients (>18 years old) identified as having a suspected GBM underwent surgical resection or biopsy were recruited from the Queen Elizabeth II Health Sciences Centre following written, informed consent. All surgeries were carried out under general anesthesia with standard monitoring of vitals, neuronavigation and sterile surgical technique. Tumour resections were completed through a craniotomy overlying the tumour region. Biopsies were completed via a small craniotomy or burrhole with stereotactic guidance, at the discretion of the attending neurosurgeon. Whole blood samples were obtained longitudinally from just prior to the initiation of surgery (*n* = 10), 18 ± 1 days post-surgery (*n* = 6), and at the time of clinically defined progression (*n* = 5). Only samples from patients with confirmed histopathological diagnosis of GBM (2021 WHO) were subsequently analyzed. Plasma was processed as per guidelines from the International ­Society of Extracellular Vesicles (ISEVs).^75^ All samples undergo visual inspection for hemolysis prior to being ­biobanked, if hemolysis is suspected the sample is discarded. After thawing and before pre-clearing at 3000× g for 15 minutes the same is followed. Blood was collected in Vacutainer EDTA-tubes (BD). Control non-cancer plasma was obtained from Innovative Research Inc. (Novi, Michigan, United States). Blood was processed within 2 hours of collection by centrifugation at 1500× g for 15 minutes at 22 °C. The plasma fraction was stored at −80 °C. All methods were carried out in accordance with the Canadian Research Tri-Council policy on ethical conduct for research involving humans (https://ethics.gc.ca/eng/policy-politique_tcps2-eptc2_2018.html). Patients included is this study had to have pathology verified GBM (Grade 3 or 4) and be *IDH-WT*. Both male and female patients were recruited, however this study is underpowered to detect differences in sex variables and was not explored as part of this paper. As this was not a clinical trial, patients were not randomized, and investigators were not blinded to whether a sample was a control or GBM sample.

### GBM Tumour Volume Assessment

The volume of pre-surgical and post-surgical gadolinium-enhancing tumour tissue was measured on T1-weighted magnetic resonance imaging (MRI) scans, using semi-automated, intensity-based image segmentation. T1-hyperintense blood products seen on unenhanced T1-weighted images were digitally removed from gadolinium-enhanced T1-weighted images and were not included in the measurement of gadolinium-enhancing tumour tissue. Triplicate measurements were made by a single observer, and median values were recorded. Tumours without visible gadolinium enhancement were assigned a volume of 0.

### Vn96-Mediated EV Isolation and Protein or RNA Extraction

Plasma was thawed at room temperature and pre-cleared at 3000× g for 15 minutes. Peptide-affinity capture of EVs was performed following well established protocols.^42^^,^^43^^,^^46^^,^^76^ EV-RNA was extracted with the miRVana miRNA isolation kit (Invitrogen) following manufacturer’s protocol for total RNA. EV-Protein was precipitated from the organic fraction using acetone and solubilized with 8 M urea, 0.2% SDS and 1 M Tris-HCl, pH 6.8 similar to.^20^^,^^43^ Total RNA quantity and profile was assessed using Fragment Analyzer 5200 (Agilent Technologies Inc.).

### Western Blot Analysis

EV-Protein samples (25 µg) were divided into 2 aliquots of equal volume and 1 sample prepared under reducing conditions with 10% β-mercaptoethanol and the other under non-reducing conditions. Samples were loaded onto Any KD Mini-Protean TGX Stain-Free Protein gels (Bio-Rad) and transferred to 0.45 µM polyvinylidene fluoride membranes. Membranes were blocked with 5% (w/v) skim milk in tris-buffered saline with 0.1% Tween-20 (TBST). Primary antibodies were prepared in TBST with 5% skim milk: Hsc-70 (1:200, Santa Cruz Biotechnology Cat# SC-7298, RRID: AB_627761), CD63 (1:200, Santa Cruz Biotechnology Cat# SC-5275, RRID: AB_627877), FLOT1 (1:1000, Cell Signaling Technology, Cat #18634, RRID: AB_2773040), CD9 (1:200, Santa Cruz Biotechnology Cat# SC-59140, RRID: AB_1120766) and GRP94 (1:1000, New England Biolabs, Cat#2104S, RRID: AB_823506). Anti-mouse IgG (1:5,000, Jackson ImmunoResearch, Cat#115-035-003, RRID: AB_10015289), and anti-rabbit IgG (1:5,000, Jackson ImmunoResearch, Cat#111-035-003, RRID: AB_2313567) secondary antibodies were used as appropriate and conjugated to horse radish peroxidase. HEK 293T cells were obtained from ATCC (Cat# CRL-3216). HEK 293T whole cell lysate protein was used as a positive control.

### sRNA Sequencing and Bioinformatics

Next-generation sequencing was performed on EV-RNA (3-10 ng) using the NextFlex sRNA sequencing kit v3 (Perkin Elmer) following manufacturers’ protocol. cDNA libraries were quantified on iSeq prior to loading on a NovaSeq 6000 (Illumina). Raw reads from sRNAseq were converted to fastq files and the adapter sequences (TGGAATTCTCGGGTGCCAAGG) were removed using TrimGalore (v0.6.5; https://github.com/FelixKrueger/TrimGalore). Alignment was performed using STAR (v2.7.0f^77^; RRID: SCR_004463) and the GRCh38 human genome with the parameters “—outFilterScoreMinOverLread 0—outFilterMatchNmin 16—outFilterMatchNminOverLread 0—outFilterMismatchNoverLmax 0.05—alignIntronMax 1—alignEndsType EndToEnd”. The resulting .bam files were passed through a counting method by chromosome location using the Bioconductor package derfinder (v1.18.9; RRID: SCR_006442^78^) Finally, the chromosome positions with counts were annotated using multiple databases such as Gencode (v38^79^) pirnaDB (v1.7.6^80^) MINT tRNA fragment database (v2.0^81^) and Mirbase (v21^82^) R statistical environment (v4.2.2) was used to calculate the variance between the normalized expressed chromosome regions read counts with trimmed mean of M-values (TMM) normalization method and differential expression (DE) analysis was performed using the Bioconductor package edgeR (v3.18.1; RRID: SCR_006442^83^) R was used to build the following complimentary figures: upset plots with upset function from ‘upsetR’; multi-dimensional scaling (MDS) plots were built with plotMDS function from ‘edgeR’ library and ggplot from ‘ggplot2’ library; and the volcano plots were built using ggplot from ‘ggplot2’library. A false discovery rate (FDR Benjamini-Hochberg adjustment) of less than or equal to 0.05 and a fold change (FC) of greater than 1 or less than −1 were considered significant for further analysis.

### Functional Analysis and Tissue Specificity of miRNA

Functional analysis of the differentially expressed (DE) mature miRNAs between control and GBM patient samples was facilitated by the miRNA enrichment analysis and annotation tool (miEAA 2.0).^84^ Over-representation analysis (ORA) was performed with the default statistical parameters (FDR Benjamini-Hochberg adjustment and significance level 0.05). Selected databases included “Localization (RNALocate)”, “Diseases (MNDR)” and “Pathways (miRWalk)”. Results of the analysis showing significantly over- or under-represented categories were exported to Excel (Microsoft) spreadsheets and ordered by the number of observed miRNAs. Tissue enrichment analysis for DE miRNA was facilitated by miRNAtissueAtlas2.^84^ For each DE miRNA (*n* = 34) the median reads per million was downloaded for the tissue panel, including brain, bone, lung, liver, lymph, bowel, muscle, heart and kidney. Median reads per million for each miRNA was divided by the maximum expression for each specific tissue and data was visualized by a heatmap.

### Analysis of RPPH1 in Tissue and Assessment of Prognostic Value

The expression of sRNA in tissue were queried from cancer and normal tissue repositories from The Cancer Genome Atlas (TCGA) and The Genotype-Tissue Expression (GTEx) databases accessed through the UCSC Xena browser (http://xenabrowser.net).^85^ To determine the expression *RPPH1* in normal tissue as compared with GBM tissue, the TCGA Target GTEx database was filtered to select from only normal brain tissue (GTEx) and *IDH-WT* GBM tissue (TCGA). *RPPH1* expression was profiled with respect to tissue type and the raw data were displayed as a violin plot. Welch’s *t*-test was used to determine statistical significance. Kaplan-Meier (KM) survival analysis was performed using the TCGA low grade glioma and GBM database. Through Xena, grade 3 and 4 GBMs were selected. Furthermore, as the TCGA database used an antiquated classification grading scheme (denoting all grade 2 *IDH-WT* tumours as low-grade gliomas and IDH mutant tumours as GBM) GBM samples with IDH1/2 mutations were excluded from analysis and Grade 2/3 IDH wild-type tumours with known defining GBM mutations (amplifications (EGFR amplifications, TERT promoter mutations, PI3KCA mutations and H3F3A mutations) were included and reviewed with Dr Croul neuropathologist (co-author).^3^^,^^86-88^ KM curve was generated to compare GBM patients with high *RPPH1* expression to those with low *RPPH1* expression. Statistical significance was displayed as a *p-*value utilizing the log-rank test. *P* < .05 was considered significant. Analysis of *RPPH1* expression between pre-surgery, post-surgery and progression was calculated using ANOVA with multiple comparisons (*p* < .05).

### Definition of Progression/Recurrence

Clinical progression was based on the opinion of the treating oncologist after discussion at multi-disciplinary rounds and this was based on MRI changes (increase size of residual or new gad enhancement, new perfusion deficits and increased symptoms), sometimes it took a second MRI in a short interval to confirm progression vs psuedoprogression before that clinical decision occurred.

## Results

### Patient Cohort Characteristics

Age and sex of control patient’s plasma samples, along with baseline age, sex and select tumour characteristics of 10 GBM patients are shown (Supplementary Table S1). The mean age of patients with tumours at the time of surgery was 65 years, and 8 patients were male. All tumours were *IDH-WT* GBM. Half of the tumours were found to have methylated *MGMT* promoter regions (Supplementary Table S1).

### sRNAs are Differentially Expressed between Control and GBM Plasma EVs

**Figure 1.**
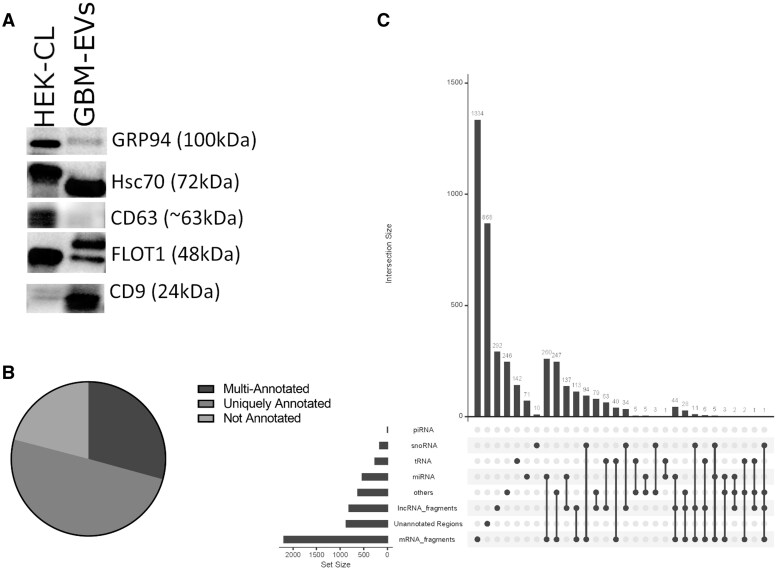
(A) Western blot analysis demonstrating EVs isolated from pooled GBM-EVs utilizing Vn96 peptide affinity have canonical markers of EVs including Hsc70, CD63, FLOT1 and CD9. The non-EV marker GRP94 is detected at low levels. HEK 293T total cell lysate (HEK-CL) was used as a positive control. Distribution of multi-annotated, uniquely annotated and not annotated RNA reads upon sequencing control and GBM samples (B); upset plot illustrating the distribution and frequency of uniquely annotated RNA sequences and multi-annotated sequences in plasma stratified by type of annotated small RNA. The columns are sorted according to a decreasing number of uniquely annotated RNA, double-annotated RNA and multi-annotated RNA with 3 or more different annotations (C).

**Figure 2.**
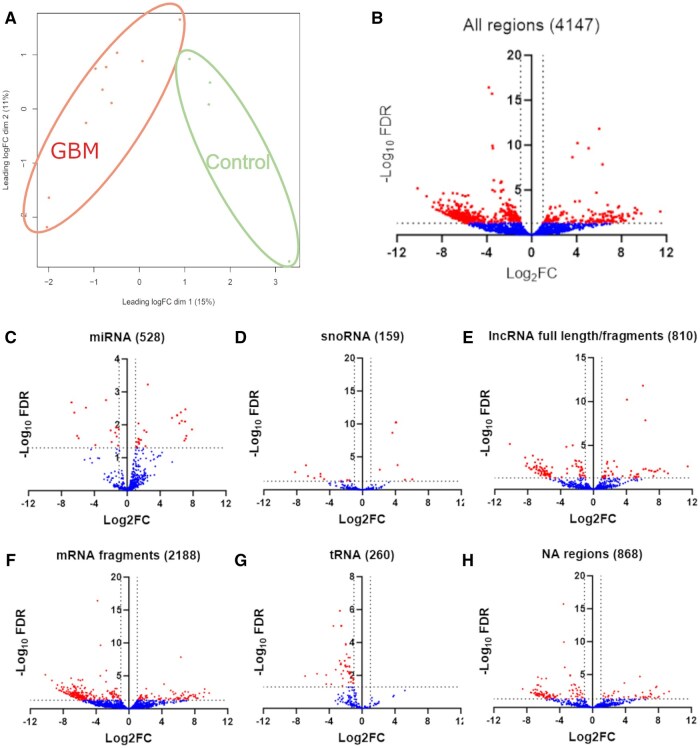
Small RNA sequencing of plasma EV contents shows significant differences when comparing controls to GBM samples. Multi-dimensional scaling plot demonstrating the degree of similarity/dissimilarity amongst GBM samples and control samples (A). Volcano plots with data points depicting differently expressed sRNA as labelled above the volcano plot with FDR < 0.05 (−log_10_FDR = 1.3) and log_2_FC > 1 or < −1 (B-H).

Following the ISEV guidelines, we confirmed the capture of EVs by Vn96 in GBM plasma, by detecting canonical EV-protein markers using Western blot analysis.^89^ As demonstrated in Figure 1A, we detected canonical EV-protein markers Hsc-70, CD63, CD9 and FLOT1 in the pooled GBM EV sample and low amounts of the non-EV protein GRP94. This demonstrates the capture of EVs from plasma of GBM patients, similar to previously reported.^42^^,^^43^^,^^46^^,^^76^ Sequencing and bioinformatic analysis of sRNA isolated from plasma EVs of controls and those from GBM patients, identified 4147 regions mapped to the human genome. Many of the regions were uniquely annotated regions, although multi-annotated and non-annotated regions were also identified (Figure 1B). The distribution and frequency of uniquely annotated and multi-annotated regions stratified by the type of annotated sRNA is shown (Figure 1C). Most uniquely annotated regions were mRNA fragments, followed by lncRNA fragments, and a smaller proportion consisted of uniquely annotated tRNA, miRNA, and snoRNA. Most multi-annotated regions showed shared sequences with mRNA fragments, followed by lncRNA (Figure 1C). Considering uniquely annotated and multi-annotated miRNA, there were 542 miRNAs sequenced. MDS plot shows control samples appearing to cluster distinctly from the GBM samples, suggesting dissimilarity between these 2 groups (Figure 2A). No correlations were observed between peri-operative medications, MGMT methylation status and molecular subtype (Supplementary Figures S1 and S2). DE analysis was performed and identified 789 regions, that passed statistical parameters, namely, FDR  < 0.05 (−log_10_FDR = 1.3) and a FC of log_2_ > 1 or < −1 (Figure 2B). To better understand the complexity of DE regions, volcano plots were generated for the most relevant annotated sRNA species including miRNA (Figure 2C), snoRNA (Figure 2D), lncRNA full length/fragments (Figure 2E), mRNA fragments (Figure 2F), tRNA ­(Figure 2G) and for regions that have no known annotation (Figure 2H).

### Functional Analysis of Differentially Expressed miRNA Reveal Associations with GBM and Pathways Involved in GBM Pathogenesis

**Figure 3.**
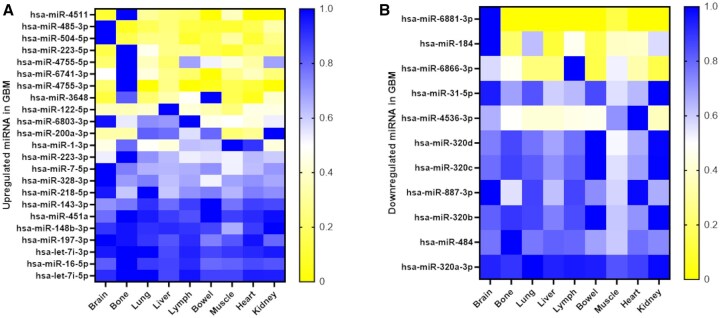
Tissue enrichment of DE miRNA. Normalized tissue expression level from TissueAtlas of 23 upregulated (A) and 11 downregulated (B) miRNA found comparing controls and GBM.

Of these 528 miRNAs, 34 mature miRNAs were found to be significantly DE between control and GBM samples (Supplementary Table S2). Considering the direction of FC, 22 mature miRNAs were found to be enriched in GBM, while 12 were found to be depleted in GBM (Supplementary Table S2). Utilizing miEAA 2.0, ORA indicated significant over-representation of the 34 mature miRNAs in categories and subcategories associated with GBMs, cancer, and EVs (Supplementary Table S3). Additionally, ORA revealed over-representation of the 34 mature miRNAs in categories associated with hallmarks of GBM pathogenesis including angiogenesis, proliferation/invasion (Supplementary Table S4), and their relevant signaling pathways. For the full ORA output see Supplementary Excel Sheet S1. To understand the tissue origin of our DE miRNA, we utilized Tissue Atlas 2.0 (TissueAtlas (uni-saarland.de)). As shown in Figure 3, a number of our DE miRNA have enriched expression in brain (miR-485-3p, miR-504-5p, miR-6881-3p and miR-184) and bone (miR-4511, miR-223-5p, miR-4755-5p, miR-6741-3p and miR-4755-3p).

### SnoRNA, lncRNA and Y-RNA are Differentially Expressed between Control and GBM plasma-EVs

Also detected were DE sRNA species other than miRNA, for which pathway analysis was unavailable. As shown in ­Figure 2D, 7 snoRNA were found enriched in GBM whereas 9 were found depleted. For example, members of the *SNORD3* family (*SNORD3A*, *3B*, *3C*, *3D*), *SNORD14B*, *SNORD42A* and *SNORD89* were found to be enriched in GBM. In addition, 54 enriched and 103 depleted annotated regions to lncRNA full length/fragments were detected ­(Figure 2E). Since the library preparation kit used was designed for sRNA most of the regions annotated for lncRNA (and similarly mRNA) were only fragments of the entire transcript, as reported by others.^52^^,^^90^^,^^91^ However, in some cases, where the full-length functional lncRNA transcript is short, a large portion of the transcript was detected and statistically significant differences in these lncRNA were sequenced. For example, for lncRNAs *MALAT1* and *RPPH1*, large portions of the transcript were sequenced (∼40% and ∼100% respectively) and these were enriched in GBM.

**Figure 4.**
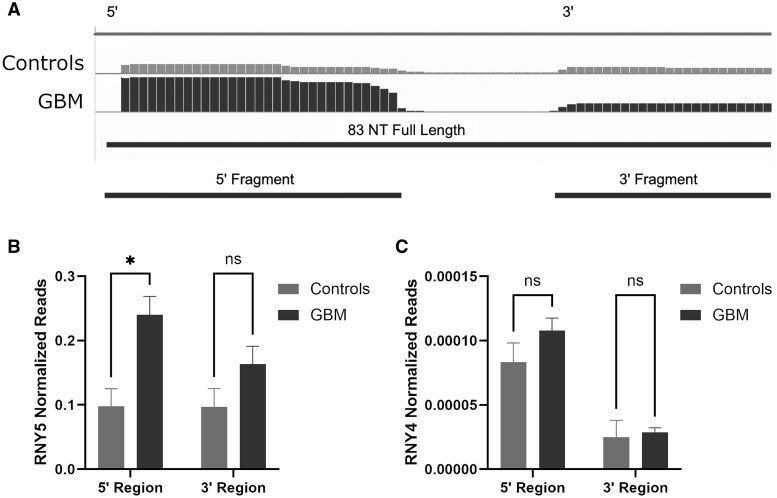
Y-RNA, RNY4 and the 5’ fragment of RNY5 are significantly enriched in GBM plasma EVs compared to control samples. Utilizing IGV, we demonstrated the 31-nucleotide, 5’ effector fragment of RNY5, known to be involved in cancer pathogenesis is highly enriched in GBM compared to the 3’ fragment, whereas controls show similar frequency of 3’ and 5’ fragments (A). Specifically, the 31-nucleotide (nt) 5’ RNY5 region is enriched in GBM compared to controls. The 3’ region is similar between GBM and controls (B). There is no difference in the proportion of 5’ and 3’ fragments of RNY4 between GBM and controls (C). *p < .05. Abbreviation: ns, non-significant.

In certain cases, particularly in cancer, DE of fragments of sRNA may be clinically important. Like other studies,^92^^,^^93^ where cell-death effector fragments of *RNY5* and tumour-suppressive fragments of *RNY4* were detected in EVs from cancer cells, we detected enrichment in the 5’ (31 nt) effector region of *RNY5* in GBM (Figure 4A and B). *RNY4* was also found to be enriched in GBM plasma-EVs, but unlike *RNY5,* there was no significant difference in the proportions of *RNY4* fragments seen between GBM and controls ­(Figure 4C). Examples of additional DE lncRNA and Y-RNA are shown in Supplementary Table S5.

### Post-Surgical Trends in sRNA Identified as Significant in GBM vs control Samples Identifies Important Biomarkers Including RPPH1 for Monitoring Disease and Signaling Progression

**Figure 5.**
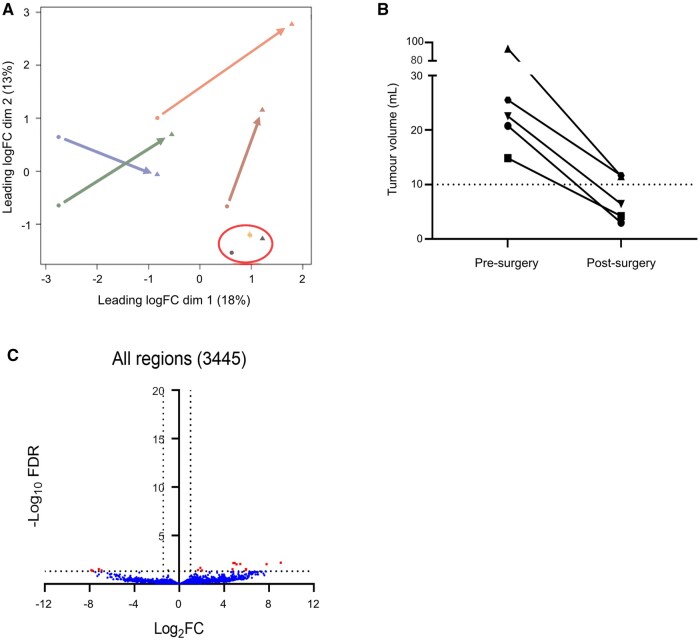
Small RNA sequencing comparing pre- and post-surgery samples from GBM patients. MDS plot demonstrating the degree of similarity/dissimilarity of sRNA profiles between pre-surgery and post-surgery samples (A). Tumour volume (mL) measured on T1-enhanced MRI imaging pre- and post-surgery for matched small RNA sequencing profiles (B). Interestingly, the tumours with the highest volume of residual had the least directional change suggesting sRNA signature may be sensitive to residual disease. Volcano plot with data points depicting differently expressed RNA as labelled above the volcano plot with FDR < 0.05 (−log_10_FDR = 1.3) and log_2_FC > 1 or < −1 (C).

**Figure 6.**
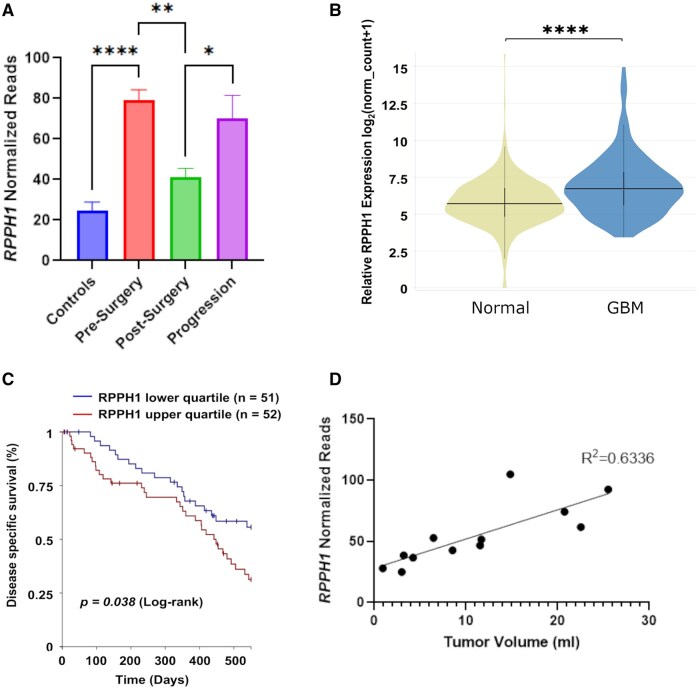
*RPPH1* expression from GBM plasma EVs serves as a candidate biomarker. *RPPH1* is DE in GBM (*n* = 10) relative to control plasma EVs (*n* = 4), shows significant reduction in expression following surgery (*n* = 6), and shows significant elevation in expression with clinically defined progression of GBM (*n* = 5) (A). *RPPH1* expression is significantly greater in GBM tissue when compared to normal tissue and higher expression is associated with worsened prognosis in GBM patients. Violin plot comparing relative *RPPH1* expression levels between normal brain tissue (*n* = 1141, GTEx database) and GBM (*n* = 144, TCGA database) as generated using Xena (B). Log Rank Kaplan-Meier curve demonstrating worse disease specific survival for the upper quartile vs lower quartile of *RPPH1* expression as generated using Xena and querying TCGA glioblastoma databases at 500 days (see Materials and Methods) (C). (D) Simple linear regression analysis between *RPPH1* normalized reads and tumor volume (mL). *p < .05; **p < .01; ****p < .0001.

We sought to see changes in sRNA profiles following surgery for GBM resection and interpret any differences in pre- and post-surgery profiles. We performed MDS to visualize global changes and observed heterogenous changes in expression levels of sRNA when comparing matched pre- and post-surgery samples (Figure 5A). Two pairs of samples did not show a marked change and were found clustered together as highlighted in the red circle. Interestingly, these 2 patients showed a high volume of post-surgical residual tumour (Figure 5B). sRNA profiling of plasma-EVs appeared to be influenced by the extent of tumour remaining after surgery, with larger tumour volumes remaining post-surgery being associated with minimal changes in sRNA profiles. DE analysis, with the same statistical parameters described previously, revealed only 23 regions DE between pre- and post-surgery samples (Figure 5C). For a list of DE sRNA in pre- and post-surgery plasma-EV comparison, see Supplementary Excel Sheet S2. *RPPH1*, a known plasma EV biomarker in colorectal cancer,^53^ showed a significant reduction in expression post-surgery (*p =* .006, Figure 6A). Given *RPPH1* was significant in GBM vs control and demonstrated reduced expression post-surgery, *RPPH1* expression was queried in 5 patient plasma samples in our cohort, at the time of clinically defined progression (273 ± 29 days post-surgery; *n* = 5) after standard treatment (Figure 6A). As anticipated, *RPPH1* expression significantly increased in plasma-EV samples at clinically defined progression. The detection of *RPPH1* transcript in EVs derived from GBM cells was confirmed in U251 cells using RT-PCR (Supplementary Figure S3). Therefore, we characterize *RPPH1* as an important plasma-EV biomarker that not only identifies GBM, but also serves as an indicator for surgical resection and GBM progression.

### RPPH1 is Upregulated in GBM Tissue and Higher Expression Levels are Associated with Worse Disease Specific Survival

Given the strength of our data supporting *RPPH1* as a novel biomarker in GBM and support from the literature for *RPPH1* in other cancer subtypes, *RPPH1* expression was queried in GTEx TCGA databases for GBM, using UCSC Xena. *RPPH1* expression was greater in GBM tissue (*n* = 144) relative to normal brain tissue (*n* = 1141) (Figure 6B). Furthermore, KM survival analysis showed that high *RPPH1* tissue expression was associated with worse disease specific survival in *IDH-WT* GBM (Figure 6C). Finally, regression analysis was performed to compare plasma EV-*RPPH1* normalized reads to tumor volume (mL) and a modest positive correlation was found (*R*^2^ = 0.6336; Figure 6D). No correlation was observed between plasma EV-*RPPH1* normalized reads and the time to progression-free-survival (data not shown). RPPH1 is enriched in both plasma-EVs, and tissues of *IDH-WT* GBM patients compared to controls and higher levels of expression is associated with a worse prognosis in these patients, further strengthening its importance as a novel GBM biomarker.

## Discussion

The goal of non-invasive liquid biopsy is to identify biomarkers for GBM, which can predict the correct diagnosis of newly identified brain tumours, provide insight into relative survival or prognosis, be obtainable throughout the course of the disease to predict progression and/or response to adjunct therapies, and ultimately provide novel therapeutic targets. We profiled sRNA from Vn96-EVs isolated from GBM patient plasma sampled perioperatively and during post-surgical follow up, and demonstrated a potential unique, diagnostic sRNA signature for GBM. Although a small sample size (*n* = 10) compared to a younger normal cohort we identified lncRNA *RPPH1* as an important potential plasma EV biomarker for GBM, with expression levels informing on diagnosis, extent of GBM resection, and tumour progression in this small cohort. This preliminary data suggest that further investigation in a larger cohort of samples with age-matched controls should be undertaken to determine whether plasma EV *RPPH1* has prognostic and potentially predictive value for recurrence.

Comparative analysis of sRNA biomarkers in GBM across studies is complicated by several variables. Key among these are: (1) differences between tissue-derived and liquid biopsy (eg, blood) samples; (2) variation in EV isolation methodologies; and (3) selection of library preparation kits, which can preferentially enrich for distinct RNA species (eg, total RNA vs sRNA). The biological relevance of concordant or discordant sRNA profiles between GBM tissue and GBM-derived EVs remains incompletely understood. Notably, GBM cells are known to selectively sort sRNAs into EVs.^94^ For instance, oncogenic transformation may lead to intracellular retention of pro-oncogenic sRNAs while tumor-suppressive small RNAs are preferentially packaged into EVs and exported. However, when the primary aim is the identification of plasma-based biomarkers for GBM, the functional role of these sRNAs is not necessarily required. Plasma offers distinct advantages for biomarker discovery, including minimally invasive and repeatable sampling, as well as the potential to capture signals from the entire tumor microenvironment (TME)—something that tumor tissue alone may miss. The choice of blood fraction also impacts results; serum contains high levels of platelet-derived particles that can skew results.^95^ Additionally, library preparation methods play a critical role in shaping sRNA transcriptomic outputs. For example, oligo-dT priming enriches for polyadenylated mRNA, potentially at the expense of sRNA representation. Despite these technical and biological challenges, sRNAs consistently identified across multiple, methodologically diverse studies represent promising candidates for clinical translation as biomarkers or therapeutic targets. Abnormal miRNA expression in both cancer tissue and tumour-associated EVs has been extensively studied in GBM, revealing their influence on hallmark processes of tumour growth and progression.^72-74^

Given the large body of functional data linking miRNAs to GBM, bioinformatic tools such as miEAA can categorize these molecules by localization and disease processes. Using miEAA, our analysis found that the DE miRNAs were significantly over-represented in categories related to EV localization, brain cancer and functional cancer hallmark pathways (via miRWalk) known to drive GBM proliferation, invasion and angiogenesis.

Of the 34 DE miRNAs identified, 26 have established functional roles in GBM according to the literature and several miRNAs frequently reported in GBM EV studies, including members of the let-7 family, miR-21, miR-106, miR-130, miR-155, miR-185, miR-193, miR-210, miR-222, miR-451, miR-485, miR-486 and miR-574 are also observed in our analysis.^96^^,^^97^ Although some discrepancies with the literature are noted—likely due to methodological differences—several miRNAs, particularly let-7, miR-451 and miR-485, consistently emerge across studies. The co-localization of many of these miRNAs in EVs, coupled with their involvement in GBM-related pathways, supports the hypothesis that the sRNA EV biomarkers detected originate from the TME. Further studies isolating EVs from primary cell culture or from the tumour itself may provide more concrete evidence moving forward. While the precise cellular source—GBM cells vs supportive stromal cells—requires further investigation, the miEAA results lend confidence to a true EV signal emanating from the tumour milieu. This is further supported in the literature demonstrating GBM has a 5-fold increase in EVs compared to healthy controls.^34^^,^^35^

Comparing pre- and post-surgery plasma EV sRNA is complex and influenced by individual patient factors such as degree of resection, extent of residual tumour volume, post-surgical inflammation and kinetics of the tumour/EVs. Although post-surgical changes in plasma sRNA EVs signatures were variable across samples, intriguing our data suggest (limited by sample size to draw a definitive conclusion) there may be a thresholding effect of surgical resection on alterations in EV signatures. In our data, this effect is seen at 10 cm^3^ where EV sRNA plasma signatures did not alter with greater residual volumes. This concept aligns with GBM survival data that suggest a similar thresholding effect of residual disease and GBM survival.^98^^,^^99^ Interestingly, in a pediatric grade 4 brain tumour, medulloblastoma, 1.5 cm^3^ of tumour remaining on post-operative imaging is consistent with poor survival.^100^ Despite our data being preliminary, researchers should be aware of a potential thresholding effect when examining plasma EV data.

Y-RNAs are a conserved class of biologically active non-coding sRNAs found in EVs and implicated in cancer.^101^ Both RNY4 and RNY5 are elevated in GBM EVs versus controls but differ in processing. The 5’ (31nt) RNY5 fragment from cancer EVs induces cell death preferentially in non-cancer cells, suggesting a potential to modulate the TME to promote tumor survival.^93^ Previous GBM CSF studies identified RNY4 and RNY5 as biomarkers in CSF and glioma stem cell exosomes.^52^ Given plasma is easier to obtain, we confirm RNY4/5 as plasma EV biomarkers for GBM, with the 5’ RNY5 fragment specifically enriched and functionally relevant. In contrast, full-length RNY4 is enriched in GBM plasma EVs. The co-enrichment of RNY4, RNY5, and *RPPH1*—transcribed by RNA polymerase III (Pol III)—in GBM plasma EVs suggests a potential functional relationship.^102^  *RPPH1*, the catalytic RNA of RNase P involved in RNA ­cleavage, may mediate RNY5 processing, though studies continue.^103^^,^^104^

In our study, *RPPH1* was enriched in plasma-derived EVs from GBM patients compared to healthy controls and decreased following tumor resection, with levels rising again at clinical progression. Although the source of *RPPH1*-containing EVs in plasma is unclear, prior studies suggest most EVs in GBM originate from tumor cells within the TME.^34^ We and others have detected *RPPH1* in glioma cell line culture media.^52^ Analysis of TCGA data via XENA^85^ confirmed that *RPPH1* is significantly elevated in GBM tissue versus normal brain and correlates with poorer prognosis, supporting its association with the TME. This pattern parallels findings in colorectal cancer (CRC), where *RPPH1* is enriched in plasma exosomes and tumor tissue and linked to worse survival.^53^ Similar prognostic relevance of high *RPPH1* expression has been reported in NSCLC, gastric and breast cancers.^54^^,^^55^^,^^105^ Notably, *RPPH1* exists in spliced forms, including lnc-*RPPH1* and circ-*RPPH1*; GEO data show circ-*RPPH1* is elevated in GBM and predicts survival.^106^ Our sequencing approach did not capture circRNAs, warranting further study on lnc- and circ-*RPPH1* in GBM. *RPPH1* is ­implicated in cancer pathways such as miRNA sponging (miR-122, miR-326), promoting M2 macrophage polar­ization, WNT1/β-catenin signaling, and precursor tRNA ­cleavage.^53^^,^^54^^,^^102^^,^^107^^,^^108^ Future work should clarify *RPPH1*’s functional role in GBM.

Our findings establish the feasibility of plasma EV sRNA profiling as a minimally invasive biomarker platform for GBM although further studies in larger cohorts are warranted to definitively determine clinical utility. We identified a novel sRNA signature distinguishing GBM patients from controls and demonstrated that plasma EV *RPPH1* levels track disease status from diagnosis through surgery and progression, offering a potential candidate biomarker for multi-modal disease surveillance. This could be examined in a prospective manner in a larger cohort versus age-matched healthy controls Given *RPPH1*’s strong association with GBM tissue expression and patient outcomes, further functional significance and potential therapeutic targeting of *RPPH1* (eg, via antisense RNA strategies) should be explored. Prospective validation of these biomarkers in larger, multi-institutional cohorts is necessary to determine their sensitivity, specificity and prognostic utility. Additionally, functional studies of *RPPH1* and other key sRNA species will illuminate their roles in GBM biology and may reveal novel therapeutic targets. Standardizing EV isolation and RNA sequencing protocols will be essential to harmonize data and accelerate clinical translation. From an epidemiologic perspective, our study contributes to the growing evidence supporting liquid biopsies as tools for cancer surveillance and early detection, with potential applications in risk stratification and personalized treatment monitoring. Plasma EV sRNA biomarkers could facilitate population-level screening and intervention strategies, ultimately improving GBM patient outcomes.

## Supplementary Material

vdaf273_Supplementary_Data

## Data Availability

The datasets generated and/or analysed during the current study are available in the NCBI Gene Expression Omnibus (GEO) repository under the accession number GSE269391.
