# Supplementary material for: Plasma extracellular vesicle sampling from glioblastoma demonstrates a small RNA signature indicative of disease and identifies lncRNA RPPH1 as a biomarker
Source: Neurooncol Adv. 2026 Jan 7;8(1):vdaf273. doi: 10.1093/noajnl/vdaf273 (PMC12883209; doi:10.1093/noajnl/vdaf273)
Supplement: vdaf273_Supplementary_Data [file vdaf273_supplementary_data.zip › Supplemental Table 4.docx]

Table 4: Significantly enriched categories related to angiogenesis obtained via ORA (miEAA 2.0) [87] for 34 DE miRNAs when comparing control to GBM patients. This demonstrates the potential role for EV-mediated miRNA transport to contribute to cell to cell communication in the TME, by regulating new blood vessel formation or proliferation and invasion.

| **Category** | **Subcategory** | **Enrichment** | **False Discovery Rate** | **Observed miRNA** |
| --- | --- | --- | --- | --- |
| Angiogenesis |  |  |  |  |
| Pathways (miRWalk) | P00005 Angiogenesis | over-represented | 0.0010 | 18 |
| Pathways (miRWalk) | P00021 FGF signaling pathway | over-represented | 0.0005 | 17 |
| Pathways (miRWalk) | P00056 VEGF signaling pathway | over-represented | 0.0023 | 14 |
| Pathways (miRWalk) | P04393 Ras Pathway | over-represented | 0.0024 | 14 |
| Pathways (miRWalk) | P00057 Wnt signaling pathway | over-represented | 0.0005 | 19 |
| Proliferation and Invasion |  |  |  |  |
| Pathways (miRWalk) | P00018 EGF receptor signaling pathway | over-represented | 0.0013 | 16 |
| Pathways (miRWalk) | P00034 Integrin signalling pathway | over-represented | 0.0029 | 16 |
| Pathways (miRWalk) | hsa04350 TGF beta signaling pathway | over-represented | 0.0067 | 14 |
| Pathways (miRWalk) | P00048 PI3 kinase pathway | over-represented | 0.0088 | 13 |
| Pathways (miRWalk) | P00012 Cadherin signaling pathway | over-represented | 0.0226 | 11 |
| Pathways (miRWalk) | WP422 MAPK Cascade | over-represented | 0.0025 | 10 |
