# Supplementary material for: Plasma extracellular vesicle sampling from glioblastoma demonstrates a small RNA signature indicative of disease and identifies lncRNA RPPH1 as a biomarker
Source: Neurooncol Adv. 2026 Jan 7;8(1):vdaf273. doi: 10.1093/noajnl/vdaf273 (PMC12883209; doi:10.1093/noajnl/vdaf273)
Supplement: vdaf273_Supplementary_Data [file vdaf273_supplementary_data.zip › Supplemental Table 5.docx]

Table 5: Differentially expressed snoRNA, lncRNA, and Y-RNA between control and GBM. snoRNA, lncRNA, and Y-RNA are sorted by increasing FDR

| **Enriched In GBM plasma EVs-ID** | | **Log_2_ Fold change** | **False Discovery Rate** |
| --- | --- | --- | --- |
| snoRNA | SNORD3B | 4.06 | 6.00x10^-11^ |
|  | SNORD3C | 4.07 | 6.00x10^-11^ |
|  | SNORD3D | 4.06 | 6.0x10^-11^ |
|  | SNORD3A | 3.63 | 2.22x10^-9^ |
|  | SNORD14B | 6.05 | 0.0265 |
|  | SNORD42A | 4.26 | 0.0002 |
|  | SNORD89 | 2.10 | 0.0009 |
| lncRNA | MALAT1 | 6.77 | 0.0069 |
|  | *RPPH1* | 1.69 | 0.0007 |
|  | RP1-283E3.8 | 7.56 | 0.0092 |
| Y-RNA | RNY5 | 1.63 | 0.0012 |
|  | RNY4 | 1.29 | 0.0170 |
| **Depleted in GBM plasma EVs-ID** | |  |  |
| snoRNA | SNORA62 | -6.67 | 0.0002 |
|  | SNORD64 | -8.19 | 0.0021 |
|  | SNORD67 | -5.11 | 0.0037 |
|  | SNORA5C | -6.63 | 0.0086 |
|  | SNORD111 | -5.10 | 0.0095 |
|  | SNORA79B | -5.86 | 0.0166 |
|  | SNORD11 | -4.67 | 0.0233 |
|  | SNORD44 | -1.86 | 0.0334 |
|  | SNORD49A | -1.57 | 0.0355 |
|  | SNORD32A | -2.60 | 0.0440 |
| lncRNA | RP11-306O13.1 | -8.29 | 0.0002 |
|  | LINC02067 | -6.91 | 0.0020 |
|  | CTD-2651B20.7 | -2.04 | 0.0024 |
|  | CTD-2651B20.6 | -2.04 | 0.0025 |
|  | HELLPAR | -6.45 | 0.0081 |
|  | RP1-236J16.2 | -5.89 | 0.0085 |
|  | NORAD | -5.99 | 0.0135 |
|  | TAGAP-AS1 | -5.63 | 0.0145 |
|  | GPR176-D | -5.96 | 0.0231 |
|  | CYTOR | -5.40 | 0.0293 |
