# Supplementary material for: Plasma extracellular vesicle sampling from glioblastoma demonstrates a small RNA signature indicative of disease and identifies lncRNA RPPH1 as a biomarker
Source: Neurooncol Adv. 2026 Jan 7;8(1):vdaf273. doi: 10.1093/noajnl/vdaf273 (PMC12883209; doi:10.1093/noajnl/vdaf273)
Supplement: vdaf273_Supplementary_Data [file vdaf273_supplementary_data.zip › Supplemental figures.docx]

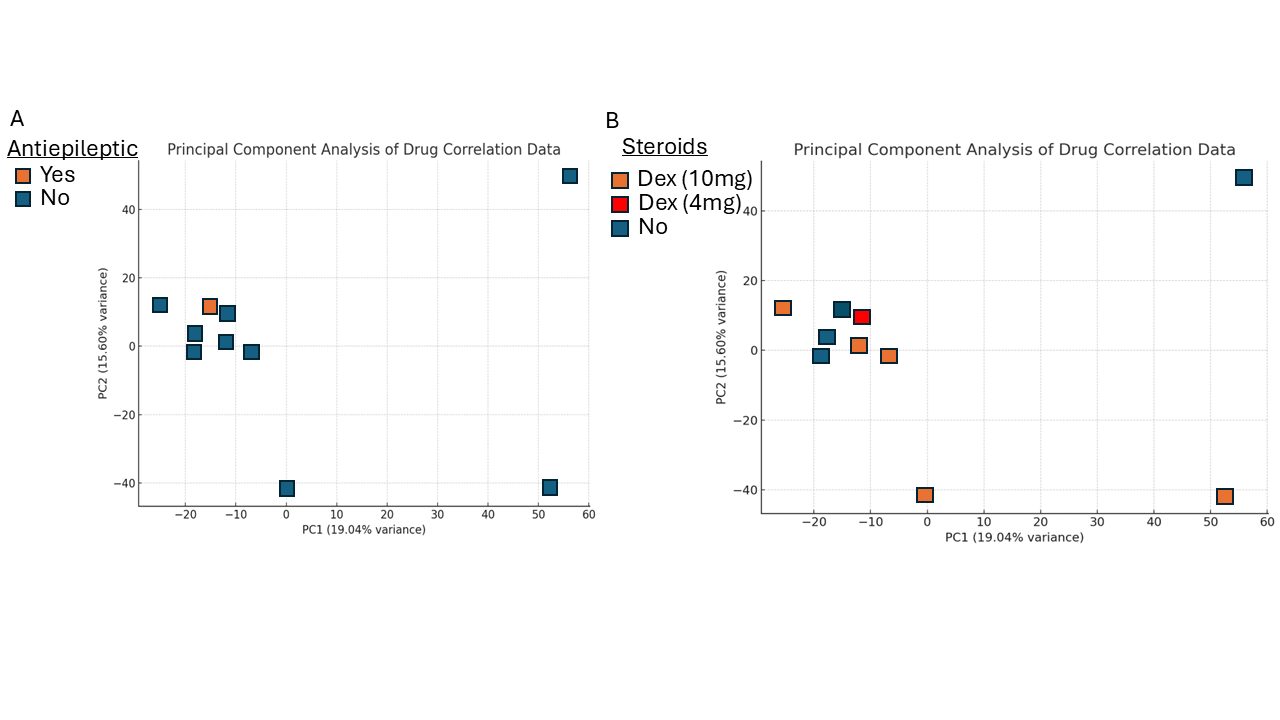


Figure 1. Principal component analysis considering patient drug factors antiepileptic (A) and steroids (B).


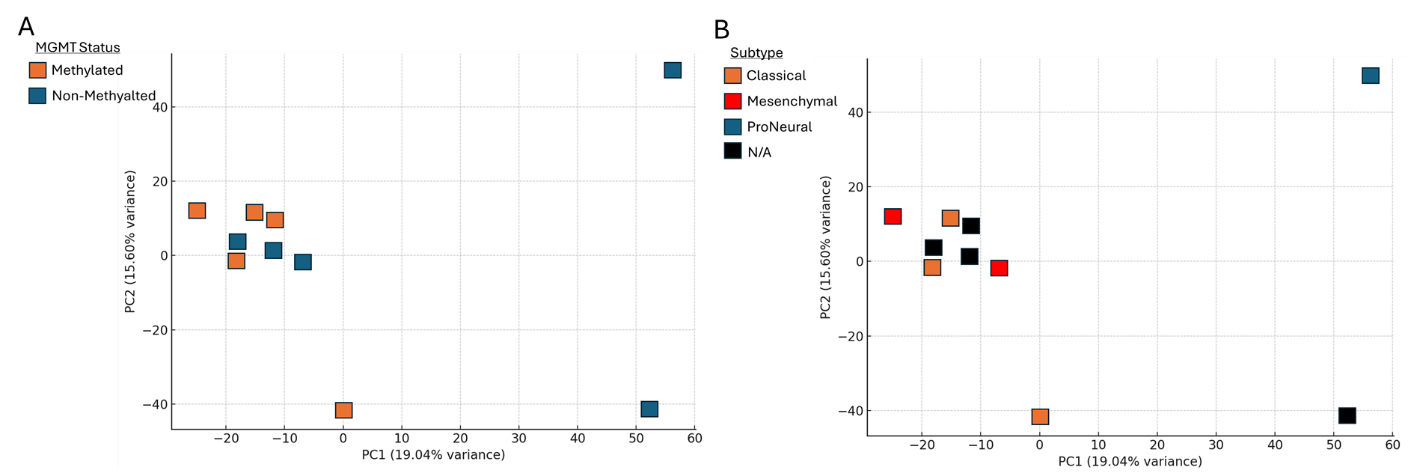

Figure 2. Principal component analysis considering MGMT status (A) and molecular subtype (B).


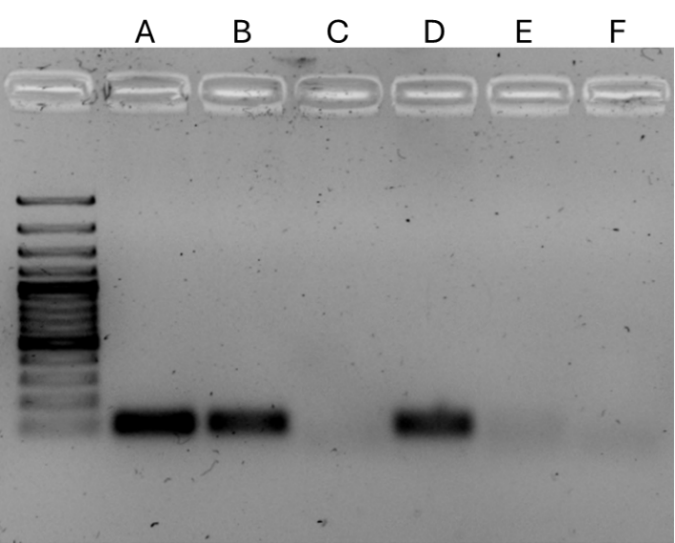


Figure 3.  RT-PCR for *RPPH1 in EV-RNA extracted from HEK cell culture media (+ve control; A), U251 cell culture media (B), non-conditioned cell culture media (C), and a GBM patient plasma (D). Included as controls are a negative RT (omission of RT enzyme, E) and H_2_O in place of cDNA (F).*
