# Supplementary material for: Plasma extracellular vesicle sampling from glioblastoma demonstrates a small RNA signature indicative of disease and identifies lncRNA RPPH1 as a biomarker
Source: Neurooncol Adv. 2026 Jan 7;8(1):vdaf273. doi: 10.1093/noajnl/vdaf273 (PMC12883209; doi:10.1093/noajnl/vdaf273)
Supplement: vdaf273_Supplementary_Data [file vdaf273_supplementary_data.zip › Supplemental Table 1.docx]

Table 1: Important patient and tumour characteristics of GBM patients and commercial, control plasma samples.

| **GBM IDH WT (n = 10)** | | **Controls (n = 4)** |
| --- | --- | --- |
| Age at diagnosis, years |  |  |
| Mean (SEM) | 65 (1.6) | 49 (3.5) |
| Median (range) | 65 (57 – 74) |  |
| Sex, no. |  |  |
| Males (% of total) | 8 (80%) | 3 (75%) |
| Females (% of total) | 2 (20%) | 1 (25%) |
| Histopathology, no. |  |  |
| Glioblastoma | 10 | N/A |
|  |  |  |
| MGMT status |  |  |
| Methylated | 5 | N/A |
| Unmethylated | 5 | N/A |
| IDH mutation status |  |  |
| Mutant | 0 | N/A |
| Wildtype | 10 | N/A |
| Tumour region |  |  |
| Frontal | 2 | N/A |
| Temporal | 7 | N/A |
| Parietal | 0 | N/A |
| Occipital | 1 | N/A |
| Steroids |  |  |
| Dexamethasone (10 mg) at Induction | 5 | N/A |
| Dexamethasone (4 mg) 2 days prior | 1 | N/A |
| None | 4 | N/A |
| Antiepileptic |  |  |
| None | 9 | N/A |
| Keppra 500 bid | 1 | N/A |
| Anesthetic |  |  |
| Propofol/Remifentanil | 10 | N/A |
