# Supplementary material for: Plasma extracellular vesicle sampling from glioblastoma demonstrates a small RNA signature indicative of disease and identifies lncRNA RPPH1 as a biomarker
Source: Neurooncol Adv. 2026 Jan 7;8(1):vdaf273. doi: 10.1093/noajnl/vdaf273 (PMC12883209; doi:10.1093/noajnl/vdaf273)
Supplement: vdaf273_Supplementary_Data [file vdaf273_supplementary_data.zip › Supplemental Table 2.docx]

Table 2: Differentially expressed mature miRNA between control and GBM. Mature miRNAs are sorted by increasing FDR.

|  | **Enriched in GBM plasma EVs-Mature ID** | **Log_2_ Fold change** | **False Discovery Rate** |
| --- | --- | --- | --- |
|  | hsa-miR-451a | 2.502 | 0.001 |
|  | hsa-miR-200a-3p | 7.071 | 0.003 |
|  | hsa-miR-4511 | 6.511 | 0.004 |
|  | hsa-miR-3648 | 6.057 | 0.005 |
|  | hsa-miR-6803-3p | 5.448 | 0.006 |
|  | hsa-miR-218-5p | 6.614 | 0.008 |
|  | hsa-miR-223-3p | 1.365 | 0.009 |
|  | hsa-miR-6741-3p | 7.868 | 0.014 |
|  | hsa-miR-197-3p | 2.062 | 0.015 |
|  | hsa-miR-122-5p | 2.334 | 0.017 |
|  | hsa-miR-504-5p | 7.175 | 0.021 |
|  | hsa-miR-4755-3p | 7.099 | 0.026 |
|  | hsa-miR-143-3p | 1.354 | 0.026 |
|  | hsa-miR-1-3p | 1.445 | 0.028 |
|  | hsa-miR-328-3p | 1.675 | 0.029 |
|  | hsa-miR-4755-5p | 6.938 | 0.030 |
|  | hsa-miR-148b-3p | 1.168 | 0.033 |
|  | hsa-miR-16-5p | 1.436 | 0.037 |
|  | hsa-miR-485-3p | 2.221 | 0.045 |
|  | hsa-let-7i-5p | 1.127 | 0.045 |
|  | hsa-let-7i-3p | 1.127 | 0.045 |
|  | hsa-miR-7-5p | 1.204 | 0.049 |
|  | **Depleted in GBM plasma EVs-Mature ID** |  |  |
|  | hsa-miR-4536-3p | -6.768 | 0.002 |
|  | hsa-miR-31-5p | -5.014 | 0.003 |
|  | hsa-miR-6866-3p | -6.427 | 0.004 |
|  | hsa-miR-223-5p | -1.330 | 0.012 |
|  | hsa-miR-320b | -1.330 | 0.012 |
|  | hsa-miR-320a-3p | -1.018 | 0.014 |
|  | hsa-miR-320d | -2.022 | 0.015 |
|  | hsa-miR-320c | -1.456 | 0.017 |
|  | hsa-miR-6881-3p | -6.093 | 0.022 |
|  | hsa-miR-887-3p | -5.899 | 0.026 |
|  | hsa-miR-184 | -3.872 | 0.040 |
|  | hsa-miR-484 | -1.110 | 0.042 |
