# Supplementary material for: Plasma extracellular vesicle sampling from glioblastoma demonstrates a small RNA signature indicative of disease and identifies lncRNA RPPH1 as a biomarker
Source: Neurooncol Adv. 2026 Jan 7;8(1):vdaf273. doi: 10.1093/noajnl/vdaf273 (PMC12883209; doi:10.1093/noajnl/vdaf273)
Supplement: vdaf273_Supplementary_Data [file vdaf273_supplementary_data.zip › Supplemental Table 3.docx]

Table 3: Significantly enriched categories related to localization and disease obtained utilizing ORA (miEAA 2.0) [87] for 34 DE miRNAs when comparing control to GBM. Importantly, this demonstrates with functional annotation and pathway analysis of our DE miRNAs, added confidence in the sRNA signature (especially the miRNA contribution), which are over-represented in subcategories for EVs and GBM.

| **Category** | **Subcategory** | **Enrichment** | **False Discovery Rate** | **Observed miRNA** |
| --- | --- | --- | --- | --- |
| Localization (RNALocate) | Microvesicle | over-represented | 0.000074 | 28 |
|  | (hsa-miR-451a; hsa-miR-200a-3p; hsa-miR-4511; hsa-miR-3648; hsa-miR-218-5p; hsa-miR-223-3p; hsa-miR-223-5p; hsa-miR-197-3p; hsa-miR-122-5p; hsa-miR-504-5p; hsa-miR-4755-3p; hsa-miR-143-3p; hsa-miR-1-3p; hsa-miR-328-3p; hsa-miR-148b-3p; hsa-miR-16-5p; hsa-miR-485-3p; hsa-let-7i-5p; hsa-let-7i-3p; hsa-miR-7-5p; hsa-miR-31-5p; hsa-miR-320b; hsa-miR-320a-3p; hsa-miR-320d; hsa-miR-320c; hsa-miR-887-3p; hsa-miR-184; hsa-miR-484) | | | |
| Diseases (MNDR) | Cancer | over-represented | 0.000401 | 27 |
|  | (hsa-miR-451a; hsa-miR-200a-3p; hsa-miR-4511; hsa-miR-218-5p; hsa-miR-223-3p; hsa-miR-223-5p; hsa-miR-197-3p; hsa-miR-122-5p; hsa-miR-504-5p; hsa-miR-4755-3p; hsa-miR-143-3p; hsa-miR-1-3p; hsa-miR-328-3p; hsa-miR-4755-5p; hsa-miR-148b-3p; hsa-miR-16-5p; hsa-miR-485-3p; hsa-let-7i-3p; hsa-miR-7-5p; hsa-miR-31-5p; hsa-miR-320b; hsa-miR-320a-3p; hsa-miR-320d; hsa-miR-320c; hsa-miR-887-3p; hsa-miR-184; hsa-miR-484) | | | |
| Diseases (MNDR) | Glioblastoma | over-represented | 0.000001 | 26 |
|  | (hsa-miR-451a; hsa-miR-200a-3p; hsa-miR-4511; hsa-miR-218-5p; hsa-miR-223-3p; hsa-miR-223-5p; hsa-miR-197-3p; hsa-miR-122-5p; hsa-miR-504-5p; hsa-miR-143-3p; hsa-miR-1-3p; hsa-miR-328-3p; hsa-miR-148b-3p; hsa-miR-16-5p; hsa-miR-485-3p; hsa-let-7i-5p; hsa-let-7i-3p; hsa-miR-7-5p; hsa-miR-31-5p; hsa-miR-320b; hsa-miR-320a-3p; hsa-miR-320d; hsa-miR-320c; hsa-miR-887-3p; hsa-miR-184; hsa-miR-484) | | | |
| Diseases (MNDR) | Malignant glioma | over-represented | 0.000074 | 22 |
|  | (hsa-miR-451a; hsa-miR-200a-3p; hsa-miR-218-5p; hsa-miR-223-3p; hsa-miR-223-5p; hsa-miR-197-3p; hsa-miR-122-5p; hsa-miR-504-5p; hsa-miR-143-3p; hsa-miR-1-3p; hsa-miR-328-3p; hsa-miR-148b-3p; hsa-miR-16-5p; hsa-miR-485-3p; hsa-let-7i-5p; hsa-miR-7-5p; hsa-miR-31-5p; hsa-miR-320b; hsa-miR-320a-3p; hsa-miR-320c; hsa-miR-184; hsa-miR-484) | | | |
| Localization (RNALocate) | Exosome | over-represented | 0.000043 | 16 |
|  | (hsa-miR-451a; hsa-miR-200a-3p; hsa-miR-218-5p; hsa-miR-223-3p; hsa-miR-197-3p; hsa-miR-122-5p; hsa-miR-143-3p; hsa-miR-328-3p; hsa-miR-148b-3p; hsa-miR-16-5p; hsa-let-7i-5p; hsa-miR-7-5p; hsa-miR-31-5p; hsa-miR-320a-3p; hsa-miR-887-3p; hsa-miR-484) | | | |
